# Data Informing Governing Body Resistance-Training Guidelines Exhibit Sex Bias: An Audit-Based Review

**DOI:** 10.1007/s40279-023-01878-1

**Published:** 2023-06-29

**Authors:** Anurag Pandit, Thai Binh Tran, Meg Letton, Emma Cowley, Mitchell Gibbs, Michael A. Wewege, Amanda D. Hagstrom

**Affiliations:** 1 Faculty of Medicine and Health, School of Health Sciences, University of New South Wales, Sydney, NSW Australia; 2 Faculty of Medicine and Health, School of Medical, Indigenous and Health Sciences, University of Wollongong, Wollongong, NSW Australia; 3 Department of Exercise and Sport Science, The University of North Carolina at Chapel Hill, Chapel Hill, NC USA; 4 Centre for Pain IMPACT, Neuroscience Research Australia, Sydney, NSW Australia

## Abstract

The objective of this review was to examine the ratio of female and male participants utilised in data informing consensus statements and position stands in the field of resistance training (RT). In order to achieve this objective, we conducted an ‘audit’ style review. We accessed three databases: SPORTDiscus, MEDLINE and Google Scholar utilising the following search terms: resistance or strength training AND consensus statements or position statements/stands. Eligibility criteria included consensus statements and position stands for RT in youth, adults and older adults. In this paper we have used the term ‘female’ to describe biological sex. Gender is a social construct and often describes roles and behaviours that society assigns to men or women. In this paper we have used the term ‘women’ to describe gender. Reference lists from each guideline were screened with the number of male and female participants extracted from each study. We also extracted data on the gender of the authors of the statements. We located 11 guidelines encompassing a total of 104,251,363 participants. Youth guidelines were comprised of 69% male participants. There were 287 studies that included both sexes, 205 male-only and 92 female-only studies. Adult guidelines were comprised of 70% male participants. There were 104 studies that included both sexes, 240 male-only and 44 female-only studies. Older adult guidelines were comprised of 54% female participants. There were 395 studies that included both sexes, 112 male-only and 83 female-only studies. Women authors comprised 13% of all authors of position stands and consensus statements. These results demonstrate an under-representation of females and women as both participants and as authors. It is imperative to ensure that data informing governing body guidelines and consensus statements are representative of the population they aim to inform. If this is not possible, guidelines should clearly state when their data and recommendations are based primarily upon one sex.

**Supplementary Information:**

The online version contains supplementary material available at 10.1007/s40279-023-01878-1.

## Key Points

**Table:** 

Current youth and adult governing body resistance-training guidelines use a heavily disproportionate volume of male data to inform recommendations.
Older adult guidelines have a more even distribution of data from both sexes, including slightly more females than males.
It is imperative to ensure that data informing governing body guidelines and consensus statements are representative of the population that the data aim to inform.
Authorship on consensus statements also demonstrates a large gender disparity, with only 13% of all authors being women. Further, 91% of all first authors were men.

## Introduction

Resistance training (RT) has documented benefits across the lifespan; for example, it is effective for improving muscular strength and hypertrophy in healthy adults [1–5], improving functional capacity in older adults [6], and increasing bone mineral density in populations with osteopenia or osteoporosis [7]. In paediatric populations, RT is safe with appropriate programming and has additional benefits including injury prevention, improved physical literacy, and improvements in motor skills, strength, speed, and power [8, 9]. RT has grown in popularity in recent years, with participation now common among athletes, youth, those with chronic diseases, and in those who were previously sedentary.

With the increasing participation in RT, there has been an expansion in the publication of consensus statements, with a particular emphasis on individual populations such as older adults [10, 11] and youth [12–18]. We have also seen the publication of numerous consensus statements relating to exercise training for a variety of chronic diseases, which often have sections devoted to them in RT guidelines [19–24].

At present, one area in which a targeted consensus statement is yet to be developed relates to RT recommendations that differ by sex. Our current understanding around whether males and females require differing exercise prescriptions is limited, both with respect to improving sports performance and in terms of general health benefits. Females exhibit physiological and morphological differences when compared to males [25]. Between sexes, physiological differences in body fat percentage and distribution, oxygen-carrying capacity, metabolic responses and biomechanics may result in marked differences in response to and recovery from RT [25–27]. Females also differ across the lifecycle with influences of the menstrual cycle, menopause and pregnancy altering physiology and the associated benefits of RT [28–31]. These differences in physiology may necessitate the need for sex-specific recommendations in some facets of exercise prescription.

While we do not currently have governing body guidelines that include or emphasise sex-specific recommendations, due to the aforementioned physiological differences, it is necessary to ensure that our guidelines are utilising data from both sexes to inform their recommendations. As such, the purpose of this study was to examine the ratio of female and male participants in studies referenced in governing body position statements for RT in healthy populations.

## Methods

### Use of Terminology

We acknowledge that in much of the data presented within the guidelines, it was not possible to ascertain whether authors and participants included in each paper were referring to biological sex or gender. While a variety of definitions are utilised, *sex* is a term that refers to the biological and physiological characteristics of an individual. In this paper we have used the term ‘female’ to describe biological sex. Gender is a social construct and often describes roles and behaviours that society assigns to men or women. In this paper we have used the term ‘women’ to describe gender. These terms are related, and although different, have historically been used interchangeably in the literature. As such, we used references to both gender and sex to categorise participants and authors in this study. As we approached the research question regarding participation with a biological lens, we used sexed terminology for the primary research question. For the section pertaining to authorship, as we assumed gender based on societal norms, we used gendered terminology in this section. We have aimed not to conflate sex and gender. We also acknowledge that sex and gender are not binary; however, at present, the data are largely presented in a binary manner in the scientific research. We acknowledge that our chosen methods of classifying sex and gender based on the above terminology may have resulted in misclassification of some individuals.

### Study Design

This review was an ‘audit’ style review, examining the ratios of male and female participants included in RT governing body guidelines and consensus statements. Audit-based reviews are novel and at present there are no formal guidelines to follow. However, we utilised a similar methodology to a recent audit-based review in a similar field [53].

### Search Strategy

An electronic search was conducted in June 2021 within the following databases: SPORTDiscus, MEDLINE and Google Scholar. The following search terms were used: resistance or strength training AND consensus statements or position statements/stands. Searches were limited to English language between the years of 2000 and the current day. A follow-up search was conducted in August 2022 to check for recently published consensus statements.

### Inclusion and Exclusion Criteria

Governing body consensus statements and position stands for RT in youth, adults and older adults were included. The reference lists from each guideline were screened, and all references to studies that involved humans were included in the analysis. Animal studies, book chapters, unpublished data and narrative reviews were not included.

### Data Extraction

Two authors independently extracted the number of male and female participants in each study. If the sex of participants was not reported, the study was excluded from analysis. Where sample size differed between recruitment and analysis, recruitment number was preferentially utilised. We chose to utilise recruitment number due to the possibility of differential reporting between outcomes within a given manuscript; hence this approach was chosen to reduce bias in terms of decisions around the selection of sample size(s) to include in our analyses. When extracting data from systematic reviews, data were extracted when sex was clearly stated. Discrepancies in extraction were resolved by a third author.

We also extracted data pertaining to the gender of all authors on each included position statement, following the method recently used in this field by Cowan et al. [32]. When we were unable to ascertain gender, we coded that data point as unable to ascertain, and excluded that individual from analyses. We also attempted to ascertain the gender of both first and last authors.

### Statistical Analyses

Statistical analyses were performed in *R.* For each guideline we calculated the ratio of total male: female participants. We also calculated the number of studies that included both sexes, only male participants, and only female participants. Sensitivity analyses excluding studies with over 100 and 200 participants were conducted to remove the influence of epidemiological studies. This number was chosen to capture any large exercise trials; however, some smaller epidemiological studies may still have been included.

For all guidelines we calculated the ratio of male and female authors. We also provided descriptive statistics relating to the frequency of articles with varying proportions of male and female authors.

## Results

### Overall

**Fig. 1:**
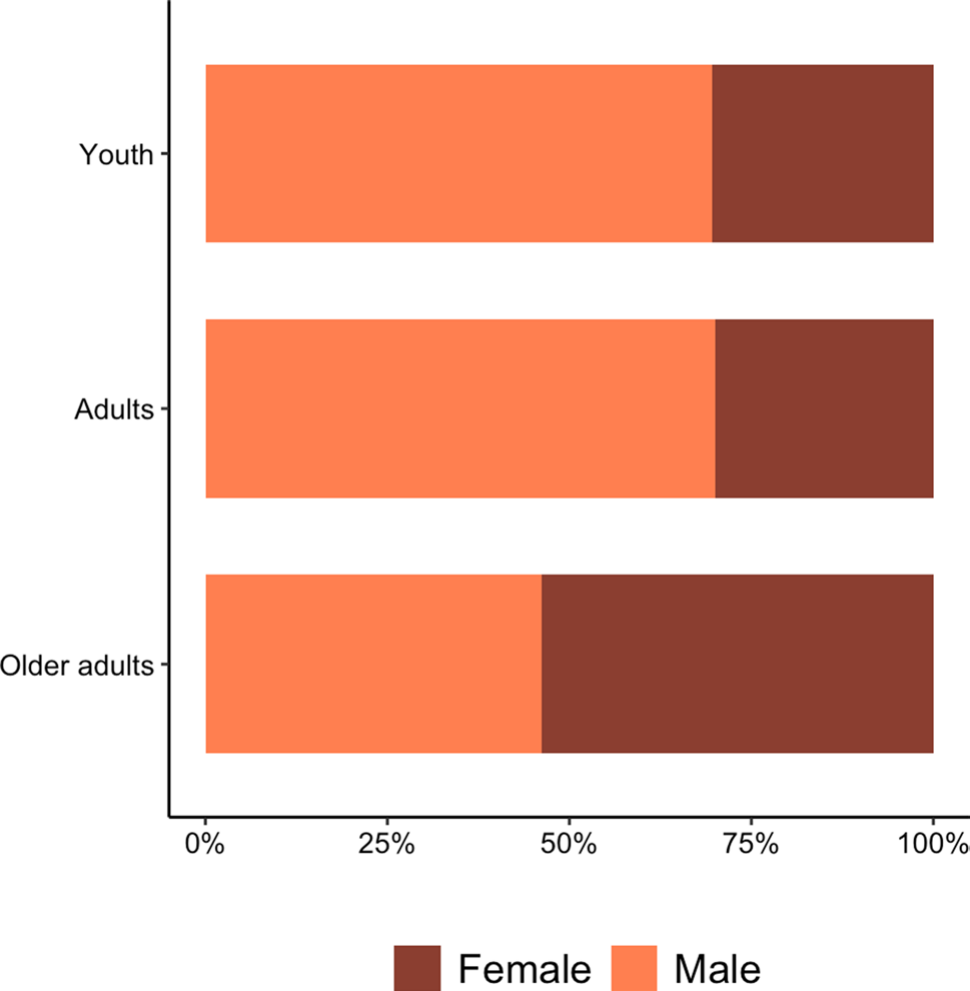
Percentage of male and female participants across youth, adult and older adult position statements and guidelines

Eleven RT guidelines published between 2004 and 2021 were identified (seven youth [12–18], two adults [33, 34], and two older adults [10, 11]). Youth guidelines included cohorts under 18 years old, while older adults included participants over 50 years old. Adult guidelines included healthy and athletic populations but did not clearly specify age ranges included in their statements. Across all guidelines, 1789 studies were eligible for inclusion, 227 of which did not report sex-related data, leaving 1562 studies for analysis. In these studies, the total number of males was 48,524,204 (47%) and the total number of females was 55,727,159 (53%). Representation by sex differed across age categories (Fig. 1).

### Youth

**Fig. 2:**
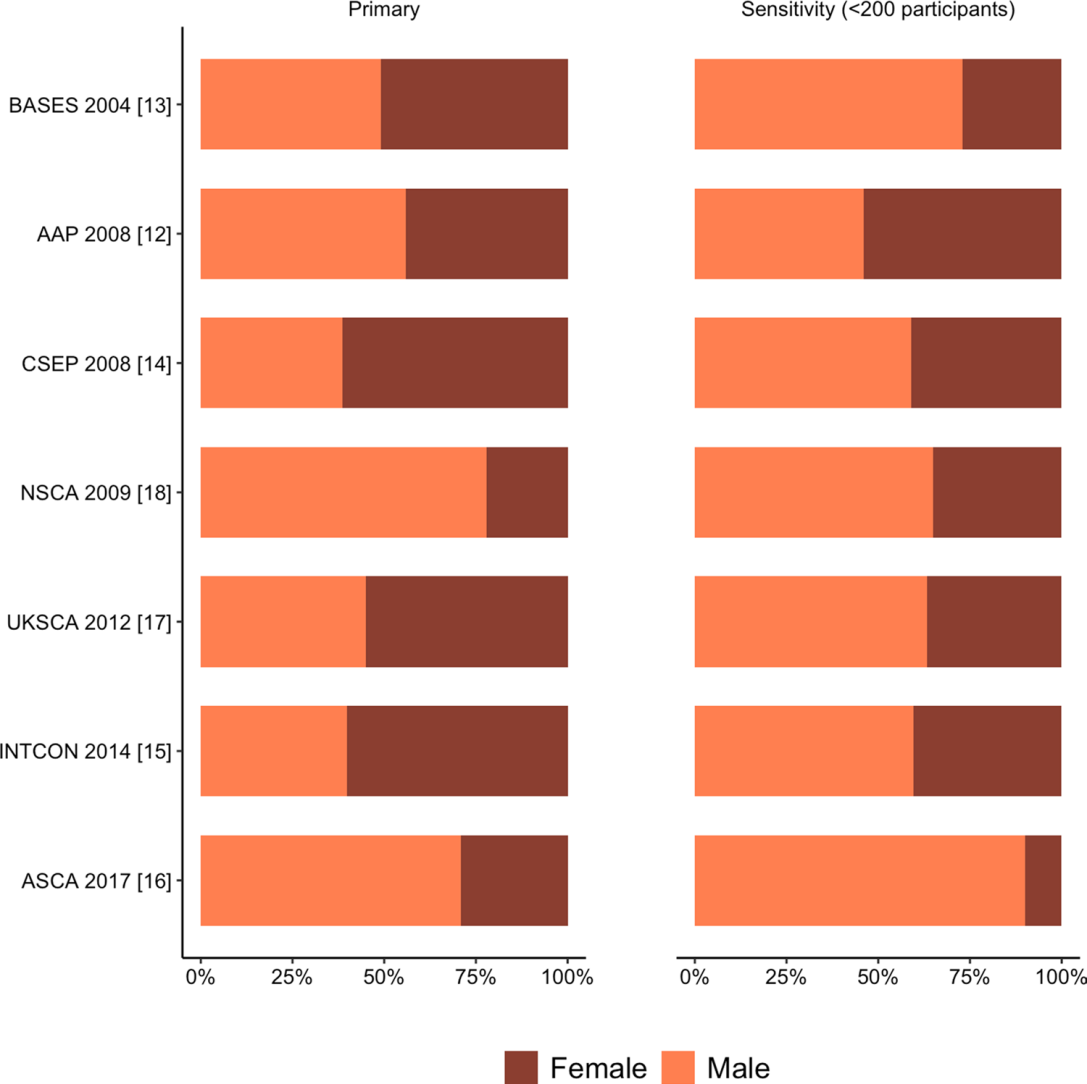
Percentage of male and female participants across individual youth position statements and guidelines. *BASES* British Association of Sport and Exercise Sciences, *AAP* American Academy of Paediatrics, *CSEP* Canadian Society for Exercise Physiology, *UKSCA* United Kingdom Strength and Conditioning Association, *INTCON* International Consensus, *ASCA* Australian Strength and Conditioning Association

Youth guidelines were comprised of 69% male participants (range 39–78%) (Fig. 2 and Online Supplementary Material (OSM) Table 1). There were 287 studies that recruited both sexes, 205 male-only studies, and 92 female-only studies. Studies that recruited both sexes had an average of 73% male participants. Studies that recruited females only averaged 440 participants per study. Studies that recruited males only averaged 84 participants per study (OSM Table 2).

Sensitivity analyses (excluding studies with greater than 100 and greater than 200 participants) did not meaningfully change results (Fig. 2 and Supplementary Table 3).

### Adults

**Fig. 3:**
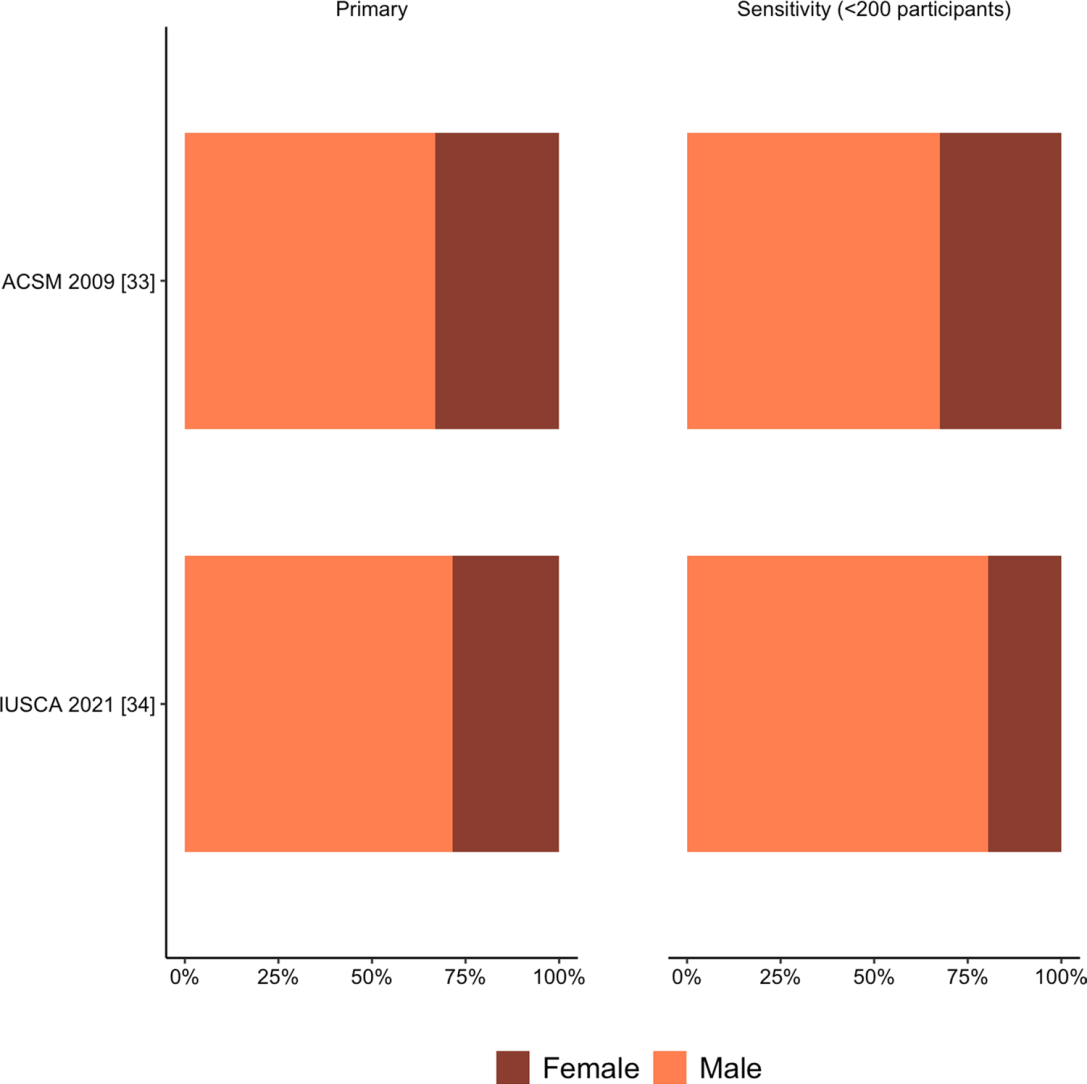
Percentage of male and female participants across individual adult position statements and guidelines. *ASCM* American College of Sports Medicine, *IUSCA* International Universities Strength and Conditioning Association

Adult guidelines were comprised of 70% (range 67–72%) male participants (Fig. 3 and OSM Table 1). There were 104 studies that recruited both sexes, 240 male-only studies, and 44 female-only studies. Studies that recruited both sexes consisted of 63% males. Female-only studies averaged 28 participants per study and male-only studies averaged 27 participants per study.

Sensitivity analyses (excluding studies with greater than 100 and greater than 200 participants) did not meaningfully change results (Fig. 3 and Supplementary Table 3).

### Older Adults

**Fig. 4:**
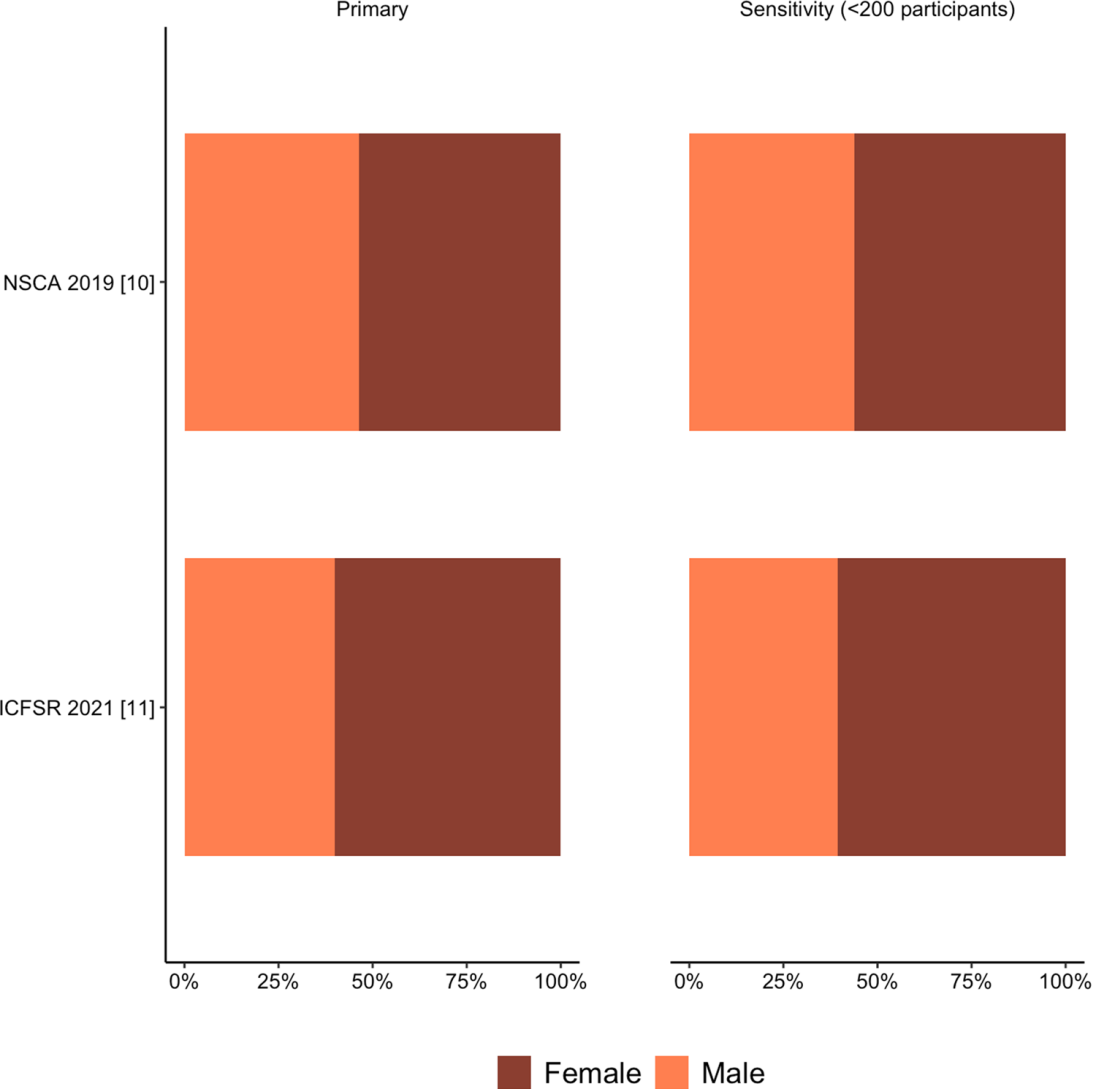
Percentage of male and female participants across individual older adult position statements and guidelines. *NSCA* National Strength and Conditioning Association, *ICFSR* International Conference on Frailty and Sarcopenia Research

Older adult guidelines were comprised of 54% (range 54–60%) female participants (Fig. 4 and OSM Table 1). There were 395 studies that recruited both sexes, 112 male-only studies, and 83 female-only studies. Studies that recruited both sexes consisted of 54% females. Female-only studies averaged 3,858 participants per study and male-only studies averaged 2,297 participants per study (OSM Table 2).

After exclusion of large cohort studies with greater than 200 participants, 57% (56–61%) of participants were female (Fig. 4 and OSM Table 3). There were 240 studies that recruited both sexes, 100 male-only studies, and 71 female-only studies. Studies that recruited both sexes consisted of 58% females. Female-only studies averaged 55 participants per study and male-only studies averaged 30 participants per study (OSM Table 4). There were no meaningful differences between the sensitivity analyses excluding studies with greater than 100 and 200 participants.

### Authorship of Position Statements

Overall, there were 121 authors for the 11 position statements. There were 103 men authors (85%), 16 women authors (13%), and two authors for whom we were unable to ascertain gender. There were three position statements with no women authors (27%), and only one with a 50% gender split. All other papers had 25% or less of authors who were women. One out of 11 first authors was a woman (9%), with ten men being first authors (91%). Three last authors were women (27%), with eight last authors being men (73%). There were seven (64%) papers with both men as first and last authors, and none with women as both first and last authors. The remaining four papers (36%) had a woman as either last or first author.

## Discussion

We found a disparity in the representation of males and females in data informing RT consensus statements and guidelines that is dependent on the age category that the statement informs. Our results showed an under-representation of females in data informing youth and adult RT consensus statements and guidelines. In contrast, guidelines informing prescription of RT for older adults included a greater representation of female participants. The reasons for these discrepancies in the volume of data presented by sex may differ in reason by age category. Further, the impacts of this disparity may also differ by group. We also found a large under-representation of women authors in governing body guidelines and consensus statements with only 13% of all authors being women, along with a commensurate under-representation of women authors in first (9%) and last authorship (27%) positions.

### Youth Statements

When examining guidelines and consensus statements relating to youth, 69% of the participants recruited were male. The reason for this large disparity is unclear. Historically, RT was actively discouraged for prepubertal youth as it was considered to have a high risk of musculoskeletal injury [35], and a perception of potential to damage bone and stunt growth. However, research demonstrated that injuries tended to occur from a lack of professional supervision or uncoordinated movement patterns [36] rather than due to RT being inherently dangerous. Interestingly, these negative associations around youth participation in RT were not sex specific. Participation in RT in youth has multiple documented benefits such as positive impacts on physical literacy, strength and body composition [37, 38]. Participation in RT by children and adolescents has also been linked with improved self-efficacy [37, 39], perceived physical ability [38, 39], self-perception [38] and confidence [39].

Given the broad and far-reaching benefits of RT in this cohort, the question remains as to why there is such a large sex-based disparity in the data presented. Historically, in the USA, female participation in sport was limited until Title IX was implemented in 1972 [40]. Title IX was part of the Education Amendments of 1972, prohibiting sex-based discrimination in any education program, inclusive of schools and universities, that received federal funding or financial assistance. Female participation in sports, such as athletics, has since risen steadily, close to male participation rates [40, 41]. Although Title IX allowed for more opportunities for girls and women to participate in sport, sport is only one facet of physical activity and exercise. Overall, female youth tend to engage in less moderate to vigorous physical activity compared to males [42], with a greater decline in the teen years [40]. Higher dropout rates and lower physical activity levels could, in part, explain the dominance of male data included in these statements. Another potential factor driving the data disparity relates to the research question of the original studies. For example, many of the studies investigating RT in youth sport tend to involve sports dominated by male participants including Australian Football, soccer, basketball and baseball [43]. Therefore, the lack of female youth in the data informing RT guidelines in this cohort may relate to a deeper systemic bias in the overall sport and exercise literature where male youth sport may be preferentially studied; however, our study was not designed to address this question so we cannot draw any conclusions. Recent research has demonstrated that female athletes are largely under-represented in sports research with most studies (70.7%) focusing on male athletes only [44].

### Adult Statements

When examining the consensus statements focusing on the adult population, we found a large disparity in the representation of data informing governing body guidelines, where approximately 70% of participants recruited in research informing guidelines were male. Like the data found in youth guidelines, the reasons for this disparity are unclear. Despite the numerous health benefits of RT, female participation levels have historically been lower than those of males, which has been attributed to historical associations of masculinity and RT, along with females expressing general feelings of discomfort in gym spaces [45]. However, a recent national survey conducted in Australia between 2001 and 2010 demonstrated that female participation in muscle-strengthening activities has increased since 2001, with females in fact more likely to report adequate muscle-strengthening activities over the previous 12 months when compared to males [46]. This historical lack of participation in RT may in part reflect the lack of data informing early guidelines; however, with contemporary data now demonstrating participation in RT does not differ by sex, there is no reason for equality not to occur moving forward. This is paramount as adults who engage in RT can expect increases in overall strength, lean body mass, and reduction in all-cause mortality [4, 5, 47–49]. Furthermore, RT can provide specific health-related benefits for females. For example, menopause has been associated with accelerated bone mineral density loss, increasing the risk of fractures and injury [50–52]. RT has been shown to have a positive effect on bone mineral density in premenopausal and postmenopausal women [53], improving health and quality of life as females transition into older adulthood. Menopause and associated health perturbations are not currently factored into the consensus statements or guidelines. The lack of inclusion of a fundamental and naturally occurring process also raises the question about what else we may be missing and what other individual sex-specific differences may exist that we are yet to elucidate. The key factor preventing data equality in these statements is likely the current lack of female specific data in the field of RT. Until this changes we cannot be sure about the importance of sex-based exercise prescriptions in this field.

### Older Adult Statements

With respect to older adults (age ≥ 50 years), our review found that 54% of all recruited participants were female. This cohort had the closest to equal representation of data from both sexes. Although the proportion of males and females represented in the older adult literature was similar, there were more male-only studies compared to female-only studies. Interestingly, in the female-only studies there were more participants per study, which increased the overall representation of females in the older adult literature.

It is unclear why this demographic had a more equal representation of participants; however, one possibility may relate to sex differences associated with ageing. For example, females tend to live longer than male equivalents in most countries [54, 55]. In addition, when compared to males, females tend to preserve body mass better with age [56], while also demonstrating a slower age-related decline of *V*O_2max_ [56]. Preservation of physical fitness and longevity of females could, in part, explain the higher participation rates in exercise studies. Another potential factor may relate to the physiological changes encountered by ageing women during the menopause and menopausal transition. The prevalence of metabolic syndrome increases with menopause [57], as does cardiovascular disease risk [58]. Menopause also has negative impacts on bone health [59]. The increase in health issues at this point in time may have influenced the representation of women in exercise-based studies as RT has been shown to benefit many of the aforementioned issues.

### Authorship of Statements

A potential contributing factor to the disparity of data observed across all consensus statements included in this review may relate to the gender of the authors of these position statements. The World Health Organization (WHO) recommends that guideline development groups be balanced in terms of gender representations [60]. We demonstrated an authorship of 13% women. A recent study examining authorship of Australian clinical practice guidelines [61] found that 47.0% of authors were men and 44.8% were women, with the gender unable to be determined for 8.2% of individuals. In the sports medicine and physiotherapy field, Cowan and colleagues demonstrated that 33% of first authors were women, and 33.2% of last authors were women [32]. We demonstrated here that 91% of first authors were men with only 9% of first authors being women. We also showed that 73% of last authors were men, compared with 27% of last authors being women. A difference in methodology relates to the authorship attribution style differences in position statements compared to original research articles. We have assumed the traditional association with first and last authors denoting seniority; however, in these instances there may be scenarios in which a chair was a first and corresponding author, and the remaining authors contributed equally. Regardless of these possibilities, our data indicate gender disparity in senior levels of authorship that is even more pronounced than in other facets of health and exercise research. The gender of authors matters as there are multiple potential flow-on effects. Firstly, sex and gender data are more likely to be reported when studies are led by women [62], and therefore, for us to continue to meaningfully reduce the sex and gender data gap, increasing the proportion of women authors would be a beneficial first step. Additionally, recent research has demonstrated that gender equality benefits all, not just women [63].

### Limitations and Considerations

Historical sociocultural influences may have influenced the data contained within the position statements included in this audit. For example, Olympic weightlifting was only added as a female sport in the 2000 Olympic Games, the same year in which CrossFit was formed. Both of these factors have greatly increased the visibility of women and RT, and in some respects are likely responsible for increased participation over recent years in all facets of RT. A disparity in historical data may simply have been due to lower levels of participation and the sociocultural influences of the time. However, if this was the case then we would expect to see a difference in the representation of males and females in the contemporary statements, which we did not observe. In the statements focused on youth (the majority of statements in our review), the publication years ranged between 2004 and 2017, and there was no clear improvement in that time. In the two adult statements published in 2009 and 2021, there was no change (or in fact a minor reduction) in the representation of women, despite the fact that RT had now been accepted and common for over 20 years at the time of the second publication. In the older adult statements, the first guideline in this review was in 2019, and as such we do not have a historical comparison available. These data indicate an ongoing and current disparity in the data that has not improved over time.

Another consideration is the influence of hormonal fluctuations. Variations in hormonal profiles across age and considerations around the menstrual cycle have historically been cited and discussed as potential variables influencing the lack of females in exercise-based research; however, it is important to note that we now have extensive guidelines by Elliot-Sale et al. [67] for researchers working with female participants. As such, this factor should not limit participation of females in contemporary sports and exercise science research. Further, best-practice methodological approaches for working with female participants should and can extend to study designs in which both sexes are recruited, rather than be applied to single-sex designs only.

A limitation of our current study relates to the inclusive nature of the research methodology. Our approach was relatively simplistic by design and by necessity; however, it may have influenced the data included in our review. For example, our current audit excluded studies that did not state the proportions of females and males, which is standard practice in literature examining sex-bias in exercise studies [64, 65]. Further, due to the difficulty in interpreting which data informed some consensus statements recommendations, in comparison to providing background data or context, we chose to include all human studies in the reference list. We believe this approach showed the breadth of literature that the authors had reviewed and likely used to draw their conclusions. We acknowledge that this may also have influenced the data; however, the direction of potential influence is unclear. In an attempt to minimise the influence of potentially large-scale epidemiological studies, we ran a secondary analysis that excluded studies with over 200 participants based on the rationale that most exercise intervention-based studies are unlikely to have cohorts over this threshold. Further, there is a possibility that the same original research studies may have been included in multiple different reviews, and hence influenced the data more than once. A further limitation is that we have not quantified the number of studies that were cited in the 11 position statements that had specific inclusion or exclusion criteria relating to sex. This information may have provided further insight as to the reason behind the data disparity. Additionally, we simply present the ratio of the sex of participants included in the statements and it is difficult to understand how the authors may have interpreted the data when writing these reviews. However, the majority of statements did not have recommendations or considerations specifically pertaining to sex or gender. The exception to this was in the youth demographic where numerous papers presented specific considerations for female youth RT.

## Conclusion

Our review found an under-representation of females in data informing youth and adult governing body RT guidelines. We also demonstrated a large under-representation of women authors and women in senior authorship positions. Unfortunately, the findings presented here are not unique to this facet of the field of sport and exercise science. Research has consistently demonstrated an under-representation of females across the breadth of original research literature with approximately 34–39% of participants being female [65, 66]. There are many potential reasons contributing to the lack of sex equity in the data presented here. We cannot discount the possible influences of historical sociocultural influences around exercise participation, particularly with respect to RT. Although some of the position statements are contemporary, the literature the authors have drawn from has potentially not yet caught up to the sociocultural norms of the present day surrounding RT participation.

While there is a need for single-sex study designs to address some research questions, there is specifically a dearth of high-quality literature relating to female exercise physiology. Further, there is still much to be learned regarding sex differences in exercise responses. Simple steps such as sex-disaggregation of data when conducting studies including both sexes will help reduce this gap and progress the field.

We encourage authors of these types of statements to be cognisant of the data that they are using to draw their inferences, and where data are not inclusive of both sexes, state this clearly in the review so the reader is able to interpret for themselves. At present, it is still largely unclear as to what degree RT participation and programming should be altered to consider sex. Until the degree of sex differences is elucidated, it is imperative to ensure that data informing governing body guidelines and consensus statements are representative of the population that the data aim to inform.

## Supplementary Information

Below is the link to the electronic supplementary material.

Supplementary file1 (DOCX 38 KB)

## Declarations

### Availability of Data and Code

Data available on request.
