# Supplementary material for: Data Informing Governing Body Resistance-Training Guidelines Exhibit Sex Bias: An Audit-Based Review
Source: Sports Med. 2023 Jun 29;53(9):1681–91. doi: 10.1007/s40279-023-01878-1 (PMC10432356; doi:10.1007/s40279-023-01878-1)
Supplement: Supplementary file 1 — Supplementary file1 (DOCX 38 KB) [file 40279_2023_1878_MOESM1_ESM.docx]

**Supplementary materials**

**Supplementary Table 1.** Participant breakdown by sex per governing body guideline

|  | **Guideline** | **No. of studies** | **Males**  **(n)** | **Females**  **(n)** | **Total**  **(n)** | **Males**  **(%)** | **Females**  **(%)** |
| --- | --- | --- | --- | --- | --- | --- | --- |
| **Youth** | AAP (2008) | 12 | 342 | 269 | 611 | 55.97 | 44.03 |
|  | ASCA (2017) | 28 | 4082 | 1676 | 5758 | 70.89 | 29.11 |
|  | BASES (2004) | 36 | 15470 | 16011 | 31481 | 49.14 | 50.86 |
|  | CSEP (2008) | 90 | 3052 | 4856 | 7908 | 38.59 | 61.41 |
|  | IntCon (2014) | 146 | 59223 | 90014 | 149568 | 39.82 | 61.41 |
|  | NSCA (2009) | 145 | 805079 | 227725 | 1032804 | 77.95 | 22.05 |
|  | UKSCA (2012) | 127 | 59223 | 72314 | 131537 | 45.02 | 54.98 |
|  | Total Youth | 584 | 946802 | 412865 | 1359667 | 69.63 | 30.37 |
| **Adults** | ACSM (2009) | 226 | 4856 | 2398 | 7254 | 66.94 | 33.06 |
|  | IUSCA (2021) | 162 | 10975 | 4368 | 15343 | 71.53 | 28.47 |
|  | Total Adults | 388 | 15831 | 6766 | 22597 | 70.06 | 29.94 |
| **Older Adults** | ICFSR (2021) | 158 | 861028 | 1294968 | 2155996 | 39.94 | 60.06 |
|  | NSCA (2019) | 432 | 46700543 | 54012560 | 100713103 | 46.24 | 53.76 |
|  | Total Older Adults | 590 | 47561571 | 55307528 | 102869099 | 46.24 | 53.76 |
| **Overall** |  | 1562 | 48524204 | 55727159 | 104251363 | 46.55 | 53.45 |

**Supplementary Table 2.** Comparison of study design by sex (individual sex or mixed cohorts) for studies included in all governing body guidelines and consensus statements

|  | **Category** | **Studies**  **(n)** | **Males**  **(n)** | **Females (n)** | **Total**  **(n)** | **Average participants/study** | **Males**  **(%)** | **Females**  **(%)** |
| --- | --- | --- | --- | --- | --- | --- | --- | --- |
| **Youth** | Combined | 287 | 929629 | 350502 | 1280131 | 4460.39 | 72.62 | 27.38 |
|  | Female | 92 | 0 | 62363 | 62363 | 677.86 | 0.00 | 100.00 |
|  | Male | 205 | 17173 | 0 | 17173 | 83.77 | 100.00 | 0.00 |
| **Adults** | Combined | 104 | 9359 | 5466 | 14825 | 142.55 | 63.13 | 36.87 |
|  | Female | 44 | 0 | 1300 | 300 | 29.55 | 0.00 | 100.00 |
|  | Male | 240 | 6472 | 0 | 6472 | 26.97 | 100.00 | 0.00 |
| **Older Adults** | Combined | 395 | 47389669 | 55078069 | 102467738 | 259411.99 | 46.255 | 53.75 |
|  | Female | 83 | 0 | 229459 | 229459 | 2764.57 | 0.00 | 100.00 |
|  | Male | 112 | 171902 | 0 | `171902 | 1534.84 | 100.00 | 0.00 |
| **Overall** | Combined | 786 | 48328657 | 55434037 | 103762694 | 132013.61 | 46.58 | 53.42 |
|  | Female | 219 | 0 | 293122 | 293122 | 1338.46 | 0.00 | 100.00 |
|  | Male | 557 | 195547 | 0 | 195547 | 351.07 | 100.00 | 0.00 |

**Supplementary Table 3.** Participant breakdown by sex per governing body guideline after exclusion of large cohort studies

|  | **Guideline** | **No. of studies** | **Males**  **(n)** | **Females**  **(n)** | **Total**  **(n)** | **Males**  **(%)** | **Females**  **(%)** |
| --- | --- | --- | --- | --- | --- | --- | --- |
| **Youth** | AAP (2008) | 11 | 161 | 189 | 350 | 46.00 | 54.00 |
|  | ASCA (2017) | 21 | 557 | 61 | 618 | 90.13 | 9.87 |
|  | BASES (2004) | 33 | 1165 | 430 | 1595 | 73.04 | 26.96 |
|  | CSEP (2008) | 84 | 1884 | 1315 | 3199 | 58.89 | 41.11 |
|  | IntCon (2014) | 106 | 3218 | 2172 | 5390 | 59.70 | 40.30 |
|  | NSCA (2009) | 122 | 4422 | 2385 | 6807 | 64.96 | 35.04 |
|  | UKSCA (2012) | 91 | 2604 | 1509 | 4113 | 63.31 | 36.69 |
|  | Total Youth | 468 | 14011 | 8061 | 22072 | 63.48 | 36.52 |
| **Adults** | ACSM (2009) | 225 | 4770 | 2284 | 7054 | 67.62 | 32.38 |
|  | IUSCA (2021) | 147 | 3269 | 790 | 4059 | 80.54 | 19.46 |
|  | Total Adults | 372 | 8039 | 3074 | 11113 | 72.34 | 27.66 |
| **Older Adults** | ICFSR (2021) | 92 | 2564 | 3931 | 6495 | 39.48 | 60.52 |
|  | NSCA (2019) | 319 | 6450 | 8229 | 14679 | 43.94 | 56.06 |
|  | Total Older Adults | 411 | 9014 | 12160 | 21174 | 42.57 | 57.43 |
| **Overall** |  | 1251 | 31064 | 23295 | 54359 | 57.14 | 42.85 |

**Supplementary Table 4.** Comparison of study design by sex (individual sex or mixed cohorts) for studies included in all governing body guidelines and consensus statements after exclusion of large cohort studies

|  | **Category** | **Studies (n)** | **Males (n)** | **Females (n)** | **Total**  **(n)** | **Average participants/study** | **Males  (%)** | **Females  (%)** |
| --- | --- | --- | --- | --- | --- | --- | --- | --- |
| **Youth** | Combined | 205 | 6785 | 4997 | 11782 | 57.47 | 57.59 | 42.41 |
|  | Female | 65 | 0 | 3064 | 3064 | 47.14 | 0.00 | 100.00 |
|  | Male | 194 | 7226 | 0 | 7226 | 37.25 | 100.00 | 0.00 |
| **Adults** | Combined | 88 | 1567 | 1774 | 3341 | 37.97 | 46.90 | 53.10 |
|  | Female | 44 | 0 | 1300 | 1300 | 29.55 | 0.00 | 100.00 |
|  | Male | 240 | 6472 | 0 | 6472 | 26.97 | 100.00 | 0.00 |
| **Older Adults** | Combined | 240 | 6016 | 8283 | 14299 | 59.58 | 42.07 | 57.93 |
|  | Female | 71 | 0 | 3877 | 3877 | 54.61 | 0.00 | 100.00 |
|  | Male | 100 | 2998 | 0 | 2998 | 29.98 | 100.00 | 0.00 |
| **Overall** | Combined | 533 | 14368 | 15054 | 29422 | 55.20 | 48.83 | 51.17 |
|  | Female | 180 | 0 | 8241 | 8241 | 45.78 | 0.00 | 100.00 |
|  | Male | 534 | 16696 | 0 | 16696 | 31.27 | 100.00 | 0.00 |
